# A Dermatologist's Ammunition in the War Against Smoking: A Photoaging App

**DOI:** 10.2196/jmir.8743

**Published:** 2017-09-21

**Authors:** Titus Josef Brinker, Alexander Enk, Martina Gatzka, Yasuhiro Nakamura, Wiebke Sondermann, Albert Joachim Omlor, Maximilian Philip Petri, Ante Karoglan, Werner Seeger, Joachim Klode, Christof von Kalle, Dirk Schadendorf

**Affiliations:** ^1^ Department of Dermatology and National Center for Tumor Diseases (NCT) University Hospital Heidelberg University of Heidelberg Heidelberg Germany; ^2^ Department of Dermatology, Venereology and Allergology University-Hospital Essen University of Duisburg-Essen Essen Germany; ^3^ German Cancer Consortium Heidelberg Germany; ^4^ Department of Dermatology and Allergic Diseases University of Ulm Ulm Germany; ^5^ Department of Skin Oncology/Dermatology Saitama Medical University International Medical Center Saitama Japan; ^6^ West German Cancer Center University of Duisburg-Essen Essen Germany; ^7^ Department of Experimental Pneumology and Allergology Saarland University Faculty of Medicine Homburg Germany; ^8^ Department of Dermatology University Hospital Magdeburg University of Magdeburg Magdeburg Germany; ^9^ Department of Internal Medicine Universities of Giessen and Marburg Lung Center; Member of the German Center for Lung Research Justus-Liebig-University of Giessen Gießen Germany; ^10^ National Center for Tumor Diseases (NCT) University of Heidelberg Heidelberg Germany; ^11^ Heidelberg Center for Personalized Oncology (DKFZ-HIPO) Heidelberg Germany; ^12^ German Cancer Research Center (DKFZ) and German Cancer Consortium (DKTK) Division of Translational Oncology University of Heidelberg Heidelberg Germany

**Keywords:** dermatology, smoking, apps, photoaging, face, skin, tobacco, tobacco cessation, tobacco prevention

## Abstract

This viewpoint reviews the perspectives for dermatology as a specialty to go beyond the substantial impact of smoking on skin disease and leverage the impact of skin changes on a person's self-concept and behavior in the design of effective interventions for smoking prevention and cessation.

Most smokers start smoking during their early adolescence, often with the idea that smoking is glamorous; the problems related to impaired wound healing, erectile dysfunction, and oral cancers are too far in the future to fathom. In contrast, for the majority of teenagers, attractiveness is the most important predictor of their own self-esteem [1].

Interventions focusing on the negative dermatologic changes due to smoking have been effective in altering behavior, both in adolescence [2-4] and young adulthood [5,6]. Skin damage due to smoking that is culturally associated with a decrease in attractiveness (ie, wrinkles, early hair loss, declined capillary perfusion, pale or grayish skin [7-9]) predominantly affects the self-concept of young people with low education [1], who are at significantly greater risk for tobacco addiction [10-12] and benefit the most from abstinence [13]. After reviewing the evidence regarding facial changes due to smoking on PubMed, we designed Figure 1 in order to extrapolate the typical appearance of a smoker’s face as frequently seen and noted by dermatologists.

**Figure 1:**
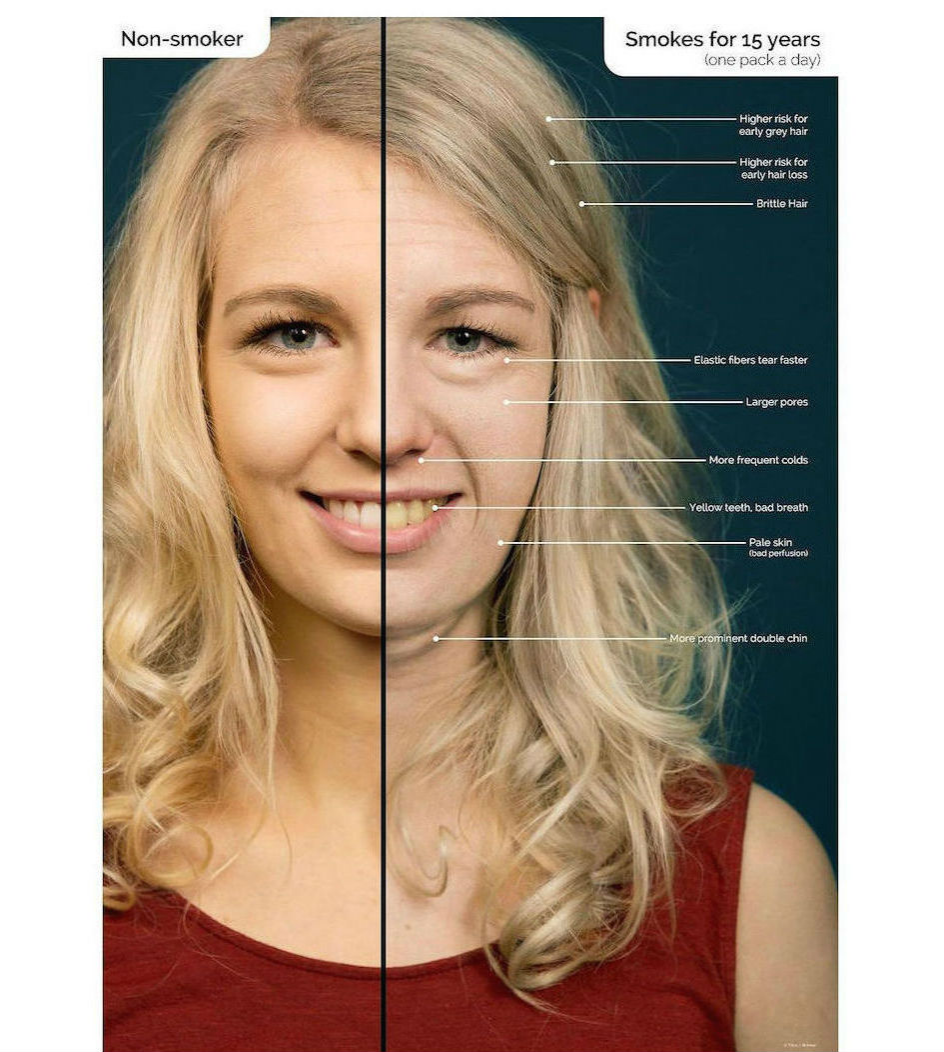
Normal aging versus effects of smoking a pack a day for 15 years.

First steps have been taken to disseminate this dermatologic knowledge on irreversible aesthetic damage to the target groups and measure its effectiveness in randomized trials (ie, via the free photoaging app Smokerface, in which a selfie is altered to predict future appearance) in Germany [3,4,14,15] and Brazil [16] with a total of more than 150,000 downloads. In addition, photoaging desktop-based interventions in France [6], Switzerland [2], and Australia [5] showed promising results that justify definitive randomized trials. The relevance of skin-based appearance for individual behavior was also confirmed in the setting of skin cancer prevention [4,17-21].

Dermatology as an interdisciplinary specialty needs to go beyond the substantial impact of smoking on skin disease [22,23] and leverage the impact of skin changes on a person’s self-concept [1] and behavior [5] in the design of effective interventions for the largest cause of preventable death and disease in the western world [24]. Future dermatologic research should focus on developing, evaluating, and optimizing new ways to implement the specialty’s superior ammunition in the war against smoking.
